# Detection of dopamine neurotransmission in “real time”

**DOI:** 10.3389/fnins.2013.00125

**Published:** 2013-07-18

**Authors:** Rajendra D. Badgaiyan

**Affiliations:** ^1^Neuroimaging and Molecular Imaging Laboratory, Department of Psychiatry, State University of New YorkBuffalo, NY, USA; ^2^Department of Radiology, Harvard Medical SchoolBoston, MA, USA; ^3^Department of Psychiatry, VA Medical CenterBuffalo, NY, USA

**Keywords:** neurotransmitter imaging, dopamine, dynamic molecular imaging, raclopride, fallypride, positron emission tomography, cognition

## Abstract

Current imaging techniques have limited ability to detect neurotransmitters released during brain processing. It is a critical limitation because neurotransmitters have significant control over the brain activity. In this context, recent development of single-scan dynamic molecular imaging technique is important because it allows detection, mapping, and measurement of dopamine released in the brain during task performance. The technique exploits the competition between endogenously released dopamine and its receptor ligand for occupancy of receptor sites. Dopamine released during task performance is detected by dynamically measuring concentration of intravenously injected radiolabeled ligand using a positron emission tomography (PET) camera. Based on the ligand concentration, values of receptor kinetic parameters are estimated. These estimates allow detection of dopamine released in the human brain during task performance.

Neuroimaging methods have made significant contribution to our understanding of the brain and behavior by localizing spatial and temporal attributes of brain processing. These methods however, have extremely limited ability to detect changes in neurochemical milieu during cognitive and behavioral processing. As a result, neurochemistry of human cognition and behavior remains mostly uninvestigated. Since neurochemicals, particularly neurotransmitters, significantly influence the brain activity, our understanding of the human brain will remain incomplete until changes in neurotransmission can be studied on a real time basis (Badgaiyan, 2011a,b).

In recent years investigators have used variations of molecular imaging methods to detect acute changes in dopamine neurotransmission during task performance (Koepp et al., 1998; Pappata et al., 2002; Alpert et al., 2003; Normandin et al., 2007; Wack and Badgaiyan, 2011). These methods exploit the competition between endogenously released neurotransmitters and their ligands for receptor occupancy. Because of this competition increasing or decreasing levels of endogenously released neurotransmitter alter the concentration of an injected ligand. Therefore, the ligand concentration measured at different brain areas indicates the level of endogenously released neurotransmitter in that area. This strategy is used to detect and map dopamine released during task performance. In dynamic molecular imaging methods volunteers receive a radiolabeled ligand prior to initiation of a task and the ligand concentration is measured dynamically during task performance using positron emission tomography (PET). Based on the concentration, values of receptor kinetic parameters are measured with the help of algorithms that account for binding of the ligand to dopamine receptors (specific binding) and to the substrates outside these receptors (non-specific binding). When dopamine is released endogenously, the specific and non-specific binding along with the values of receptor kinetic parameters are altered. The algorithms estimate these alterations to detect, map, and measure dopamine released during task-performance.

Measurement of values of receptor kinetic parameters requires collection of multiple arterial blood samples. Based on the ligand concentration in these samples, specific and non-specific binding of the ligand is estimated. Because of the need for collection of serial arterial blood samples, these experiments were performed only in a limited number of laboratories until development of the simplified reference region model (SRRM). The SRRM (Lammertsma and Hume, 1996; Gunn et al., 1997) allows measurement of values of receptor kinetic parameters without the need to collect arterial blood samples. It estimates the value in an “activated” region by comparing it to the value measured in a brain region (reference region) that contains a negligible amount of dopamine receptors (e.g., cerebellum). Because of paucity of receptors, values measured in a reference region reflect the ligand concentration outside dopamine receptors (non-specific binding). These values are used in the kinetic models such as SRRM to estimate ligand binding potential (BP), which is a sensitive index of ligand binding and a measure of receptors available to the ligand for binding. Based on the ligand binding, release of endogenous dopamine is detected and mapped. Using this method Koepp and colleagues measured BP of the ligand ^11^C-raclopride at rest (control scan) and in a second scan while the same volunteers played a video game (Koepp et al., 1998). By comparing the two BP values, they detected and mapped dopamine released during task performance (playing video game). But this method is not considered sensitive because it requires comparison of data acquired in two separate scan sessions, one at rest (control task) and the other during task performance (study task). Since the difference in the baseline dopaminergic activity during the two scan sessions cannot be accurately accounted for, the need for two separate scans compromises sensitivity of detection. This method is therefore not suitable for detection of relatively small amounts of dopamine released during performance of a cognitive task (Pappata et al., 2002; Badgaiyan, 2011a).

Thus, to enhance the sensitivity, it is necessary to acquire both control and task-induced data in the same scan session. But the data acquired using this strategy cannot be analyzed using SRRM because it assumes maintenance of a steady physiological state throughout the scan session. By including two conditions (control and study task) in a single scan, this assumption is violated. To reconcile the violation investigators used a variety of approaches. The initial approach involved comparison of the ligand BP measured during task performance with a “normalized” BP map generated using data previously acquired in a “control” experiment (Pappata et al., 2002). This approach, however, is not significantly different from the two-scan method because it also requires comparison of data acquired in two separate scan sessions. The approach implemented in our laboratory involves modification of algorithm used in SRRM to eliminate the assumption of steady state (Alpert et al., 2003; Badgaiyan et al., 2003a). The modified SRRM, called the linear extension of simplified reference region model (LE-SRRM) (Alpert et al., 2003) accepts changes in receptor kinetic parameters during a scan session and allows measurement of time dependent changes in values of these parameters.

## Single-scan dynamic molecular imaging technique

In the single scan dynamic molecular imaging technique developed in our laboratory (Alpert et al., 2003; Badgaiyan et al., 2003b) both control and test tasks are performed in the same scan session. Additionally, to allow detection of changes associated with a component of the task, instead of rest, appropriately designed task is used as a control. Thus, to detect dopamine released during emotional processing, we asked volunteers to process emotionally neutral words in the control task and emotional words in the study task.

In this method volunteers are positioned on the bed of a scanner in supine position and instructed to stay still during the entire experiment. Thereafter, a single intravenous bolus of a radiolabeled dopamine receptor ligand (either ^11^C-raclopride, 10–15 mCi; specific activity >1.0 Ci/μMol or ^18^F-fallypride, 5–8 mCi, specific activity >2.5 Ci/μMol) is administered intravenously at high specific activity in the left anticubital vein over a period of 60 s. The PET data acquisition begins immediately after the injection. At the same time, the control task is administered for 15–25 min. It is important to administer the task for about 15 min to allow the ligand to achieve steady state and to stabilize the dopamine system. After the steady state is achieved (indicated by PET counts), test task is administered for 15–25 min. Behavioral data (response time, accuracy of responses etc.) are acquired in each trial and the PET data are acquired either continuously (in list mode) or at 30 s epochs. The ligand concentration is measured dynamically in each voxel (including the reference region) at each time point during the experiment. Based on these data, kinetic models estimate values of the receptor kinetic parameters dynamically. These values are compared with those obtained in the reference region to eliminate non-specific binding and estimate specific binding at each time point. The difference in values of specific binding measured during control and study task performance indicates task-induced changes. Based on these data dopamine released during performance of a task is detected, mapped and measured in a single scan session.

The following operational equation is used to estimate time dependent changes in the ligand concentration:

$$\begin{array}{l} \text{PET}(t)=R^{*}C_{R}(t)+K_{2}^{*}\int_{0}^{i}C_{R}(u)du-k_{2a}\int_{0}^{i}\text{PET}(u)du\\ \;-\;\gamma \int_{0}^{t}v(u-T)e^{-\tau (u-T)}\text{PET}(u)du \end{array}$$

where, *C*_*R*_ is the radioligand concentration in the reference region, PET is the concentration in an “activated” voxel with specific binding, *R* is the ratio of transport rates for the binding and reference regions, *k*_2_ describes the clearance of non-specifically bound tracer from the voxel, and *k*_2*a*_ includes information about dissociation from the receptor, γ represents change in the rate of ligand displacement, *t* denotes the measurement time, *T* is the task initiation time and ν (*u*-*T*) is the unit step function.

Since the rate of ligand displacement (γ) from receptor sites closely follows endogenous dopamine release, it is considered an important parameter for detection of dopamine release (Alpert et al., 2003). As discussed earlier, the single scan method significantly enhances sensitivity of detection by eliminating confounds associated with the use of two separate scans for the control and test conditions (Alpert et al., 2003; Badgaiyan et al., 2003b).

## Use of other receptor kinetic models

In another significant development SRRM was modified by Zhou and his colleagues to allow measurement of the ligand BP and other receptor kinetic parameters during a specified time frame (Zhou et al., 2006). In this modification, called the extended simplified reference tissue model (E-SRTM), values of the receptor kinetic parameters are estimated separately during performance of the control and study tasks. Based on these estimates, dopamine released during task performance is detected and mapped.

Use of both LE-SRRM and E-SRTM in an analysis enhances the reliability because these models use different approaches to detect dopamine release. In LE-SRRM dopamine release is detected by measuring changes in the rate of ligand displacement during task performance while in E-SRTM detection is based on the comparison of ligand BP measured during performance of the control and study tasks. Further, the two models use different strategies to eliminate assumption of steady state. In LE-SRRM it is eliminated by allowing dissociation rate of the ligand to change in response to an altered synaptic level of neurotransmitter, while E-SRTM reconciles the violation of steady state by assuming that the data acquired during different task conditions (control and study) are separate datasets. Since steady state is maintained within each condition, this assumption allows use of SRRM in each dataset for measurement of BP and other receptor kinetic parameters.

When both models (LE-SRRM and E-SRTM) are used in an analysis, results are reconciled using predefined criteria. In our studies we consider a blob (>5 contiguous voxels) “activated” only if: **(a)** there is a significant change (*p* < 0.05) in values of the rate of ligand displacement estimated using LE-SRRM after task initiation; **(b)** the ligand BP (measured using E-SRTM) is significantly lower (*p* < 0.05) during performance of the study task than that during the control task; **(c)** there is a significant increase in dissociation coefficient (*k*_2*a*_) measured using E-SRTM during study task performance; and **(d)** maxima of blobs identified as “activated” by LE-SRRM and E-SRTM are located within 6 mm of each other to account for Gaussian smoothing involved in the processing. Use of these criteria results in excellent test-retest reliability (Badgaiyan and Wack, 2011).

## Selection of a ligand

Accuracy and reliability of detection in the single scan dynamic molecular imaging experiments depend on specificity and receptor kinetic properties of the ligand. Based on the receptor affinity of a ligand, dopamine can be detected either in the high or low receptor density areas of the brain. Thus, the low affinity ligand raclopride is an excellent choice for detection of dopamine neurotransmission in the high receptor density areas such as striatum. Its binding and displacement cannot be detected or measured in the low receptor density areas outside the striatum. We used this ligand to detect and map striatal dopamine released during performance of a number of cognitive, emotional and behavioral tasks (Badgaiyan et al., 2003b, 2006, 2007, 2008a; Badgaiyan, 2010; Badgaiyan and Wack, 2011). Study of dopamine neurotransmission in low receptor density areas outside the striatum requires a high affinity ligand such as ^18^F-fallypride (Badgaiyan et al., 2009) and ^11^C-FLB457 (Farde et al., 1997). High-affinity ligands are not suitable for detection of dopamine in high receptor density areas because of prolonged binding time (Mukherjee et al., 2002). We used ^18^F-fallypride to detect dopamine released outside the striatum during emotional processing in healthy volunteers (Badgaiyan et al., 2009). In this experiment we detected dopamine in the amygdala, medial temporal lobe and prefrontal cortex. Thus, depending on the ligand used, dopamine release can be detected in different brain areas.

## Use of single-scan dynamic molecular imaging technique

**Figure 1:**
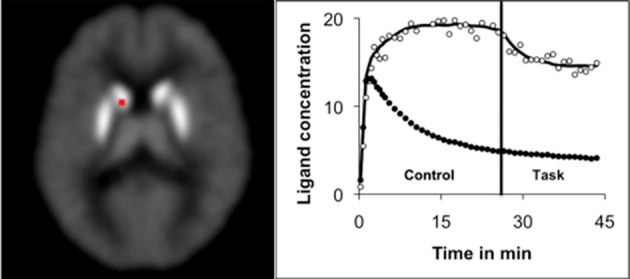
**The figure shows significant increase in the rate of ligand (^11^C-raclopride) displacement in the left caudate during performance of an executive inhibition task (Eriksen's flanker task).** As discussed in the text, the increased rate indicates task-induced release of dopamine. The time activity curve shows the ligand concentration in the right caudate (open circle) and in the cerebellum (solid circle). The cerebellum was used as a reference region. The least square fit (solid line) of the data acquired in the left caudate was drawn using LE-SRRM (see text). The curve shows increased rate of ligand displacement after initiation of the task (vertical line). In the control condition volunteers were shown congruent stimuli that did not require activation of executive inhibition. In the test condition incongruent stimuli were presented (study task). These stimuli activated the executive inhibition system. The ligand concentration is expressed as kBq/cc. Figure adapted from Badgaiyan and Wack (2011).

In the past few years, the single scan dynamic molecular imaging technique has been used by us (Badgaiyan et al., 2003b, 2007, 2008a, 2009; Badgaiyan, 2010; Badgaiyan and Wack, 2011; Badgaiyan, 2011a) and others (Christian et al., 2006; Backman et al., 2011) to reliably detect and map dopamine released during performance of a number of cognitive, behavioral, and emotional tasks in healthy volunteers (Badgaiyan et al., 2003b, 2007, 2008a, 2009; Christian et al., 2006; Badgaiyan, 2010; Backman et al., 2011; Badgaiyan and Wack, 2011) and psychiatric patients (Badgaiyan et al., 2008b; Badgaiyan, 2011a). Data obtained in these experiments provide novel insight into dopaminergic control of human brain processing. For example, in a recent study (Badgaiyan and Wack, 2011) we found dopamine release in discrete areas of the left caudate (Figure 1) during performance of a task of executive inhibition (Eriksen's flanker task), suggesting dopaminergic processing of inhibitory functions. Even though involvement of dopamine in the processing of central inhibition was suspected for several years (Casey et al., 2000), it was never demonstrated until we used the single scan dynamic molecular imaging technique. In this study we also localized clusters of striatal dopaminergic neurons involved in the processing. Further, by following cortical connections of these clusters we were able to understand how the striatal and frontal areas interact to provide effective inhibition. The cluster where dopamine release was most significant (medial aspect of the head of left caudate), receives input primarily from the orbitofrontal cortex—interestingly this input is found only on the left hemisphere (Ferry et al., 2000). Based on this input we suggested that the orbitofrontal cortex initiates inhibitory response by modulating activities of the dopaminergic system of left caudate.

The data acquired in single scan dynamic molecular imaging experiments can also be used to understand neurocognitive bases of impairments in psychiatric and neurological disorders. For example in a recent experiment (Badgaiyan et al., 2008b) we studied dysregulated dopamine neurotransmission in attention deficit hyperactivity disorder (ADHD). We conducted this study because it was unclear whether the dopaminergic system is hyperactive or hypoactive in ADHD. Studied conduced in the past used indirect measures of dopaminergic activity and found either high or low activity (Pliszka, 2005). We used dynamic molecular imaging to resolve the contradiction by mapping and measuring dopamine released in these patients during processing of an inhibitory task. In ADHD the amount of dopamine released during inhibition of unwanted responses (phasic release) was significantly enhanced (Badgaiyan et al., 2008b) but the release at rest (tonic release) was attenuated. This finding explains why indirect evidence suggests either high or low dopamine activity. Studies that have indirectly measured the phasic release have found increased activity while indirect data that are dependent on the tonic release indicated reduced activity.

## Summary

The single scan dynamic molecular imaging is an evolving technique, which expands the scope of neuroimaging research by allowing study of neurochemical change associated with cognitive or behavioral processing. The technique, however, is at the initial stages of development. Therefore, at this time it cannot be used to detect temporal sequence of events or to simultaneously detect multiple neurotransmitters. Additionally, because task sequence cannot be altered the control task always has to precede the test. It therefore introduces sequence bias. Currently, efforts are underway to resolve these limitations by developing appropriate receptor kinetic models and by modifying experimental protocol (Badgaiyan, 2011a,b).

### Conflict of interest statement

The author declares that the research was conducted in the absence of any commercial or financial relationships that could be construed as a potential conflict of interest.
